# Propofol Inhibits Cerebellar Parallel Fiber-Purkinje Cell Synaptic Transmission via Activation of Presynaptic GABA_B_ Receptors *in vitro* in Mice

**DOI:** 10.3389/fnins.2018.00922

**Published:** 2018-12-06

**Authors:** Fang-Ling Xuan, Hong-Wei Wang, Li-Xin Cao, Yan-Hua Bing, Chun-Ping Chu, Ri Jin, De-Lai Qiu

**Affiliations:** ^1^Key Laboratory of Cellular Function and Pharmacology of Jilin Province, Yanbian University, Yanji, China; ^2^Department of Physiology and Pathophysiology, College of Medicine, Yanbian University, Yanji, China; ^3^Department of Cardiology, Affiliated Zhongshan Hospital of Dalian University, Dalian, China; ^4^Department of Osteology, Affiliated Hospital of Yanbian University, Yanji, China

**Keywords:** propofol, cerebellar Purkinje cell, whole-cell patch-clamp recording, GABA_B_ receptor, parallel fiber, protein kinase A

## Abstract

Propofol is a widely used intravenous sedative-hypnotic agent, which causes rapid and reliable loss of consciousness via activation of γ -aminobutyric acid A (GABA_A_) receptors. We previously found that propofol inhibited cerebellar Purkinje cells (PC) activity via both GABA_A_ and glycine receptors *in vivo* in mice. We here examined the effect of propofol on the cerebellar parallel fiber (PF)-PC synaptic transmission in mouse cerebellar slices by whole-cell recording technique and pharmacological methods. We found that following blockade of GABA_A_ and glycine receptors activity, propofol reversely decreased the amplitude of PF-PC excitatory postsynaptic currents (PF-PC EPSCs), and significantly increased paired-pulse ratio (PPR). The propofol-induced decrease in amplitude of PF-PC EPSCs was concentration-dependent. The half-inhibitory concentration (IC_50_) of propofol for inhibiting PF-PC EPSCs was 4.7 μM. Notably, the propofol-induced changes in amplitude and PPR of PF-PC EPSCs were abolished by GABA_B_ receptor antagonist, saclofen (10 μM), but not blocked by N-methyl-D-aspartate receptor (NMDA) receptor antagonist, D-APV (50 μM). Application of the GABA_B_ receptor agonist baclofen induced a decrease in amplitude and an increase in PPR of PF-PC EPSCs, as well masked the propofol-induced changes in PF-PC EPSCs. Moreover, the propofol-induced changes in amplitude and PPR of PF-PC EPSCs were abolished by a specific protein kinase A (PKA) inhibitor, KT5720. These results indicate that application of propofol facilitates presynaptic GABA_B_ receptors, resulting in a depression of PF-PC synaptic transmission via PKA signaling pathway in mouse cerebellar cortex. The results suggest that the interaction with GABA_B_ receptors may contribute to the general anesthetic action of propofol.

## Introduction

Propofol, an emulsion formulation of 2,6-diisopropylphenol, which is a rapid-acting sedative-hypnotic medication, has been widely used for the induction and maintenance of anesthesia. The electrophysiological studies demonstrated that propofol significantly increased GABA-mediated inhibitory transmission in rat olfactory cortex slices (Collins, 1988), and reversibly enhanced the amplitude of GABA-evoked Cl^−^ currents in rat cortical neurons (Hales and Lambert, 1991). Extracellular administration of propofol evoked inward Cl^−^ currents in acutely dissociated hippocampal pyramidal neurons and depressed rat spinal cords neuronal activity through activation of GABA_A_ receptors activity (Jewett et al., 1992; Hara et al., 1994; Grasshoff and Antkowiak, 2004). The hypnotic effects of propofol are primarily attributed to the enhancement of GABA_A_ receptor function by extending GABA_A_ channels open times (Kitamura et al., 2004) and slowing desensitization (Bai et al., 1999, 2001). Whole-cell patch clamp recordings from medial entorhinal cortical neurons showed that dipropofol hyperpolarized the resting membrane potential and reduced the number of action potential firings, resulting in inhibition of neuronal excitability via activating GABA_A_ currents (Zhang et al., 2018). Under *in vivo* conditions, propofol decreased the facial stimulation-evoked spike firing activity of molecular layer interneurons through enhancement of GABA_A_ receptors activity (He et al., 2015).

However, it has been demonstrated that propofol potentiated both GABA- and glycine-induced chloride currents at small concentrations, but inhibited both GABA- and glycine-induced Cl^−^ currents at large concentrations in acutely dissociated rat spinal dorsal horn neurons (Dong and Xu, 2002). Blocking glycine receptors activity significantly attenuated the propofol-induced loss of righting reflex in rats, as well the propofol-induced current of rat hypothalamic neurons, suggesting that glycine receptors contribute to propofol-induced hypnosis (Nguyen et al., 2009). We recently demonstrated that propofol inhibited the spontaneous simple spike (SS) activity of cerebellar Purkinje cells (PCs) through activation of both GABA_A_ and glycine receptors, suggesting that propofol depressed the SS outputs of cerebellar PCs which involved in both GABA_A_ and glycine receptors activity (Jin et al., 2015). On the other hand, it has been reported that anesthetic doses of propofol failed to affect glycinergic synaptic transmission in the spinal neurons (Wakita et al., 2016).

With exception of modulation of GABA_A_ and glycine receptors, propofol may modulate NMDA and GABA_B_ receptors activity. Propofol attenuated the neurotoxic effect of glutamate exerted via activation of NMDA receptors in cultured hippocampal neurons, while augmented the NMDA-mediated effect on intracellular calcium in a cerebral ischemia model (Zhan et al., 2001). Blockade of NMDA receptor activity not only inhibited facial stimulation-evoked responses in mouse cerebellar granule cell layer, but also abolished their enhancement by propofol, suggesting that propofol enhanced granule cell layer responses via modulation of NMDA receptor (Jin et al., 2016). In addition, intravenous administration of propofol depressed the activity of nigral DA neurons, which was prevented by the selective GABA_B_-receptor antagonist, suggesting that activation of central GABA_B_ receptors may contribute to the anesthetic properties of propofol (Schwieler et al., 2003). The cerebellar PCs receive excitatory afferents through mossy fiber-granule cell-parallel fibers (PFs) and provide the sole output from the cerebellar cortex to the deep cerebellar nuclei for motor planning, execution, and coordination in their neuronal activity (Palay and Chan-Palay, 1974). Besides various excitatory postsynaptic receptors, cerebellar cortical PF-PC synapses express a wide range of presynaptic receptors, such as GABA_A_, GABA_B_, cannabinoids 1 (CB1), and NMDA receptor (Casado et al., 2002; Qiu and Knöpfel, 2007; Chu et al., 2014; Orts-Del’Immagine and Pugh, 2018). Although propofol depresses the spontaneous SS activity of cerebellar PCs via modulating GABA_A_ and glycine receptors activity, the effect of propofol on the cerebellar PF-PC synaptic transmission is currently unknown. We here examined the effect of propofol on the cerebellar PF-PC synaptic transmission in mouse cerebellar slices by whole-cell recording technique and pharmacological methods.

## Materials and Methods

### Slice Preparation

The experimental procedures were approved by the Animal Care and Use Committee of Yanbian University and were in accordance with the animal welfare guidelines of the U.S. National Institutes of Health. The permit number is SYXK (Ji) 2011-006. Cerebellar slices preparation has been previously described (Qiu and Knöpfel, 2007). In brief, adult (4–6 weeks old) ICR (Institute of Cancer Research) mice were deeply anaesthetized with halothane and decapitated quickly. The cerebellum was dissected and placed in ice-cold artificial cerebrospinal fluid (ACSF) containing (in mM): 125 NaCl, 3 KCl, 1 MgSO_4_, 2 CaCl_2_, 1 NaH_2_PO_4_, 25 NaHCO_3_, and 10 D-glucose bubbled with 95% O_2_/5% CO_2_ (pH 7.4; 295–305 mOsm). The sagittal slices of cerebellar cortex (250 μm thick) were prepared using a Vibratome (VT 1200s, Leica, Nussloch, Germany). The slices were incubated for ≥ 1 h in a chamber filled with 95%O_2_/5% CO_2_ equilibrated ACSF at room temperature (24–25°C) prior to recording.

### Electrophysiological Recordings and PF Electrical Stimulation

Whole-cell patch-clamp recordings from PC somas in cerebellar slices were visualized using a 60× water-immersion lens using a Nikon microscopy (Eclipse FN1, Nikon Corp., Tokyo, Japan). Patch electrodes contained a solution of the following (in mM): potassium gluconate 120, HEPES 10, EGTA 1, KCl 5, MgCl_2_ 3.5, NaCl 4, biocytin 8, Na_2_ATP 4, and Na_2_GTP 0.2 (pH 7.3 with KOH, osmolarity adjusted to 300 mOsm). Patch pipette resistances were 4–6 MΩ in the bath, with series resistances in the range of 10–20 MΩ. Membrane potentials and/or currents were monitored with an Axopatch 700B amplifier (Molecular Devices, Foster City, CA, United States), filtered at 5 kHz, and acquired through a Digidata 1440 series analog-to-digital interface on a personal computer using Clampex 10.4 software (Molecular Devices, Foster City, CA, United States). Cells were held in voltage clamp mode at –70 mV. Series resistance was monitored by applying voltage pulses (10 ms, 5 mV), and only cells with stable series resistance were include in the analysis. For PF electrical stimulation, a stimulating electrode containing ACSF (0.1–0.5 MΩ) was placed in the molecular layer of the cerebellar slice, and paired-current pulses (0.2 ms, 10–100 μA; duration: 50 ms) at 0.5 Hz were delivered through a glass electrode mounted on remote-controlled manipulators (MP-385, Sutter Instrument Company, Novato, CA, United States). The paired-pulse ratio (PPR) was calculated as the second EPSC amplitude over the first EPSC amplitude.

### Drug Application

Propofol, NBQX, (2,3-dioxo-6-nitro-1,2,3,4-tetrahydrobenzo [f]quinoxaline-7-sulfonamide); picrotoxin, saclofen, baclofen were bought from Sigma-Aldrich (Shanghai, China), while D-APV (D-aminophosphonovaleric acid) and KT5720 were bought from Tocris Cookson (Bristol, United Kingdom). The stock solutions of propofol (200 mM) and KT5720 (1 mM) were diluted in dimethyl sulfoxide (DMSO). The experimental concentration of DMSO was less than 0.1% throughout and did not alter or evoke any currents in separate control experiments. All other chemicals were dissolved in solution and kept in frozen in aliquots, and they were applied to the cerebellar slices at 0.5 ml/min in ACSF. In the experiments involving KT5720, application of KT5720 was started at least 10 min before recording and continuing throughout the experiments. The ACSF included picrotoxin (50 μM) and strychnine (2 μM) during all recordings to prevent GABA_A_ and glycine receptor-mediated inhibitory responses.

### Statistical Analysis

Electrophysiological data were analyzed using Clampfit 10.3 software. All data are expressed as the mean ± SEM. One-way ANOVA and Mann–Whitney-Wilcoxon test (SPSS Software; Chicago, IL, United States) was used to determine the level of statistical significance between groups of data. *P*-values below 0.05 were considered to indicate a statistically significant difference between experimental groups.

## Results

### Effect of Propofol on PF-PC Synaptic Transmission

**FIGURE 1:**
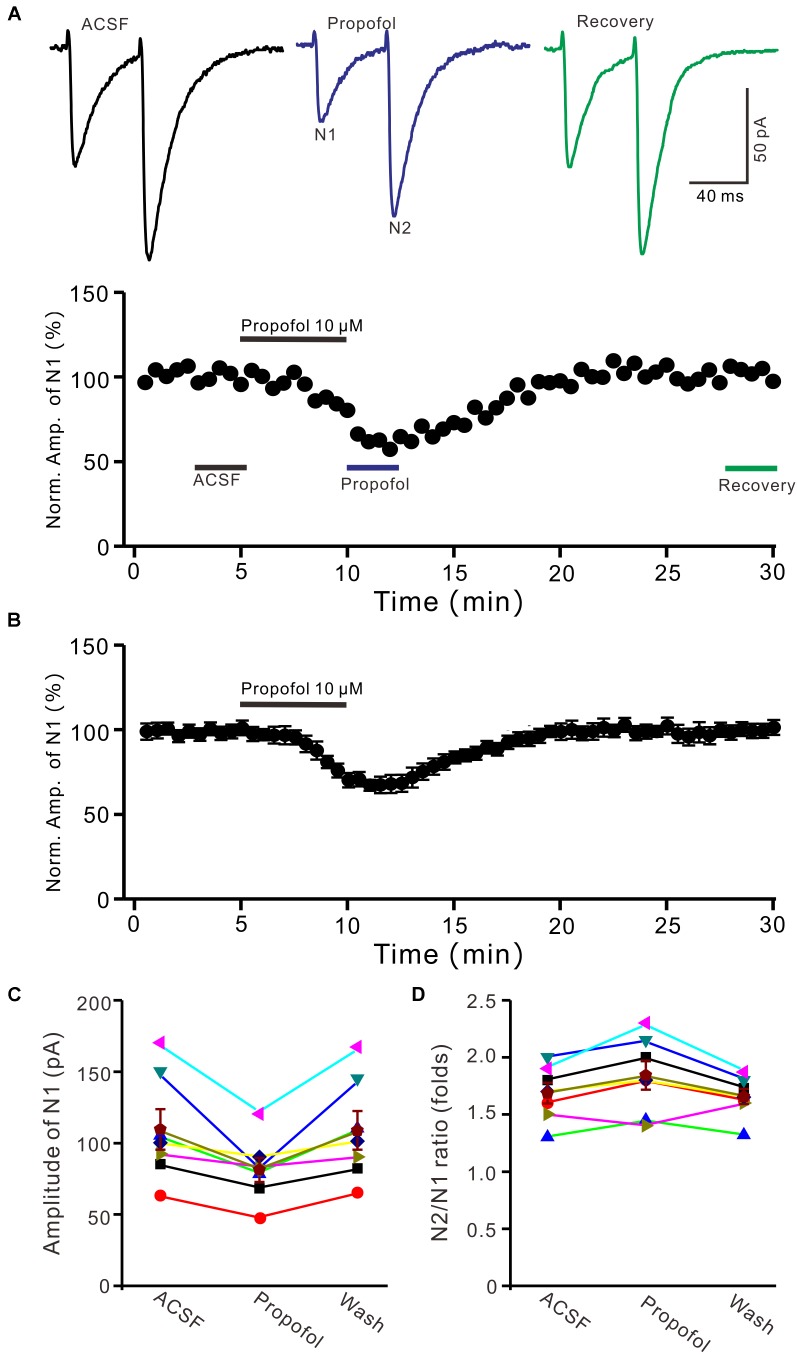
Propofol induced a decrease in amplitude and an increase in PPR of PF-PC EPSCs. **(A)** Upper, representative traces showing PF-PC EPSCs elicited by paired-pulse stimulation under control, propofol (10 μM propofol) and recovery (washout of propofol). Lower panel shows the time course of the propofol-induced changes in amplitude of N1. **(B)** Summary of data (*n* = 8) showing the time course of 10 μM propofol-induced changes in amplitude of N1. **(C)** Individual and mean (±S.E.M; *n* = 8) values are showing the amplitude of N1 in the each treatment. **(D)** Individual and mean (±S.E.M; *n* = 8) values showing the PPR in the each treatment.

**FIGURE 2:**
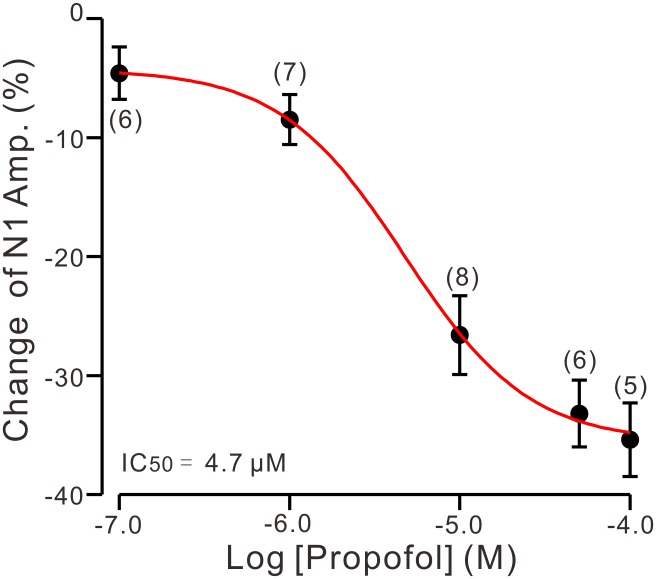
The concentration-response curve shows the propofol-induced decrease in amplitude of N1. The IC_50_ value obtained from the curve was 4.7 μM. The number of the recorded PCs tested for each concentration is indicated near the bars. Note that bath application of propofol induced a concentration-dependent decrease in amplitude of N1. Error bars indicate S.E.M.

To determine the effect of propofol on PF-PC synaptic transmission, we recorded paired PF-stimulation (0.2 ms, 10–100 μA; interval: 50 ms) evoked EPSCs of PCs under voltage-clamp recording conditions (Vh = −70 mV). Blockade of GABA_A_ and glycine receptors activity with picrotoxin and strychnine (see section Materials and Methods) increased the amplitude of PF-PC EPSCs from 95.2 ± 7.2 pA to 113.1 ± 6.5 pA (*P* = 0.003; *n* = 7; not shown). Blockingα-amino-3-hydroxy-5-methyl-4- isoxazolepropionic acid (AMPA) receptor activity with NBQX (10 μM) abolished the PF-PC EPSCs (not shown). Application of propofol (10 μM) induced a time-dependent depression of PF-PC EPSCs (Figures 1A,B) in the absence of GABA_A_ and glycine receptors activity. In the presence of propofol, the mean amplitude of N1 was 80.9 ± 8.3 pA, which was significantly weaker than that in control conditions (109.3 ± 14.2 pA; *P* = 0.0026; *n* = 8) (Figure 1C). Further, we analyzed the effect of propofol on the paired-pulse ratio (PPR; N2/N1) of PF-PC EPSCs. The mean value of PPR was 1.84 ± 0.1 in the presence of propofol, which was significantly higher than control conditions (1.68 ± 0.1; *P* = 0.0011; *n* = 8) (Figure 1D). Propofol depressed amplitude of N1 was concentration-dependent (Figure 2). The amplitude of N1 was significantly reduced by 4.6 ± 2.2% of control with 100 nM propofol (*P* = 0.032; *n* = 6), and its IC_50_ was 4.7 μM. The amplitude of N1 was reduced by 35.4 ± 3.4% of control with 100 μM propofol (*n* = 5). These results indicate that propofol induces a concentration-dependent decrease in amplitude and an increase in PPR of PF-PC EPSCs in the absence of GABA_A_ and glycine receptors activity.

### Blockade of NMDA Receptors Failed to Prevent the Propofol-Induced Depression of PF-PC EPSCs

**FIGURE 3:**
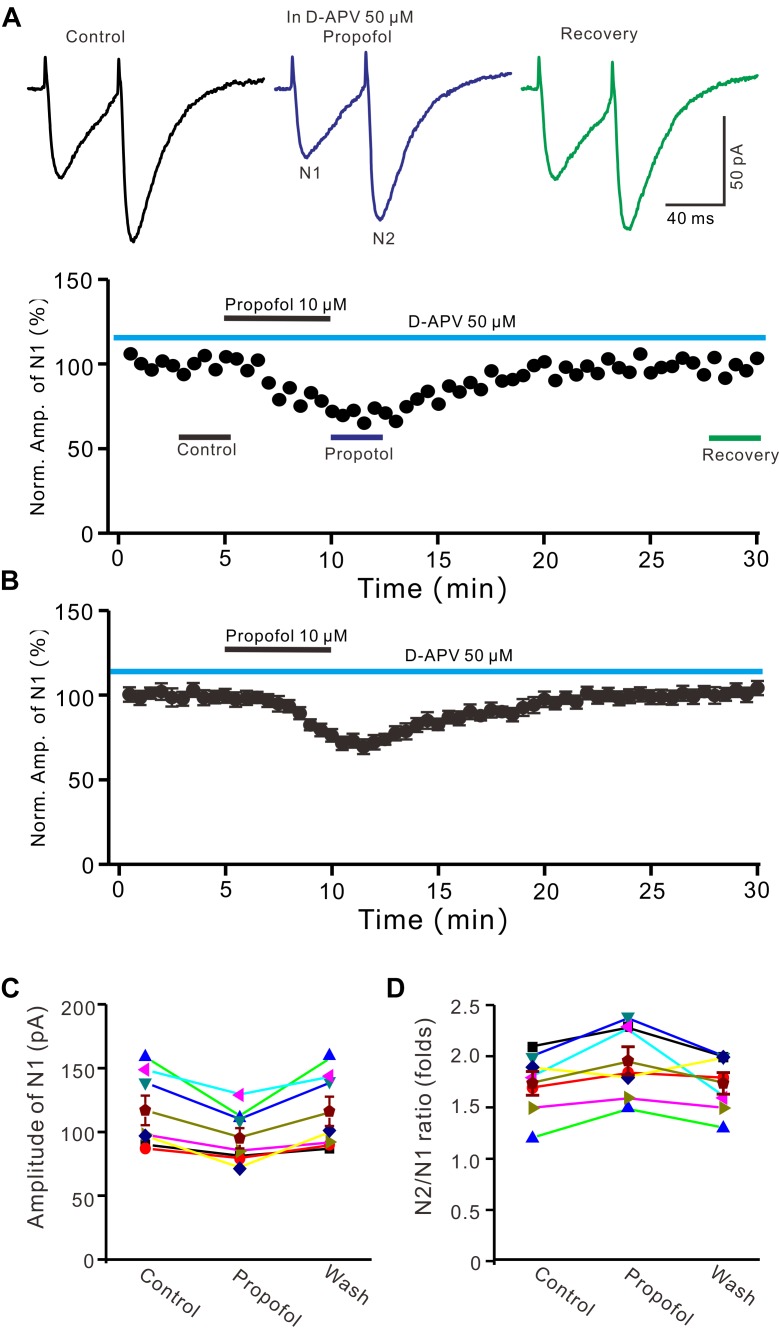
Blockade NMDA receptors failed to prevent the propofol-induced decrease in amplitude and increase in PPR of PF-PC EPSCs. **(A)** Upper, representative traces showing PF-PC EPSCs elicited by paired-pulse stimulation under control (50 μM D-APV), propofol (10 μM propofol + D-APV) and recovery (washout of propofol). Lower panel shows the time course of the propofol-induced changes in amplitude of N1 shown in upper panel. **(B)** Summary of data (*n* = 6) showing the time course of the propofol-induced changes in amplitude of N1. **(C)** Individual and mean (±S.E.M) values showing the amplitude of N1 in the each treatment. **(D)** Individual and mean (±S.E.M) values showing the PPR in the each treatment.

In cerebellar cortex, the functional NMDA receptors have been found in PFs (Casado et al., 2002), which contributed to the PF-PC presynaptic transmission and long-term plasticity (Qiu and Knöpfel, 2007; Chu et al., 2014). In addition, it has been reported that clinical concentrations of propofol inhibited NMDA receptor activity in Xenopus oocytes (Yamakura et al., 1995). We further examined whether NMDA receptors were involved in propofol-induced depression of PF-PC EPSCs in the absence of GABA_A_ and glycine receptors activity. As shown in Figure 3, in the presence of a selective NMDA receptor blocker, D-APV, additional application of propofol still induced a significant depression of PF-PC EPSCs (Figures 3A,B). The mean amplitude of N1 was 96 ± 8.1 pA, which was significant smaller than that in control conditions (118.1 ± 11.6 pA; *P* = 0.0031; *n* = 6) (Figure 3C). The mean value of PPR was 1.96 ± 0.14-fold in the presence of propofol, which was significant higher than that in control conditions (1.74 ± 0.12; *P* = 0.0023; *n* = 6) (Figure 3D). These results indicate that blockade of NMDA receptors activity does not prevent the effect of propofol on PF-PC EPSCs in the absence of GABA_A_ and glycine receptors activity.

### The Propofol-Induced Depression of PF-PC EPSCs Was Mediated by GABA_B_ Receptor

**FIGURE 4:**
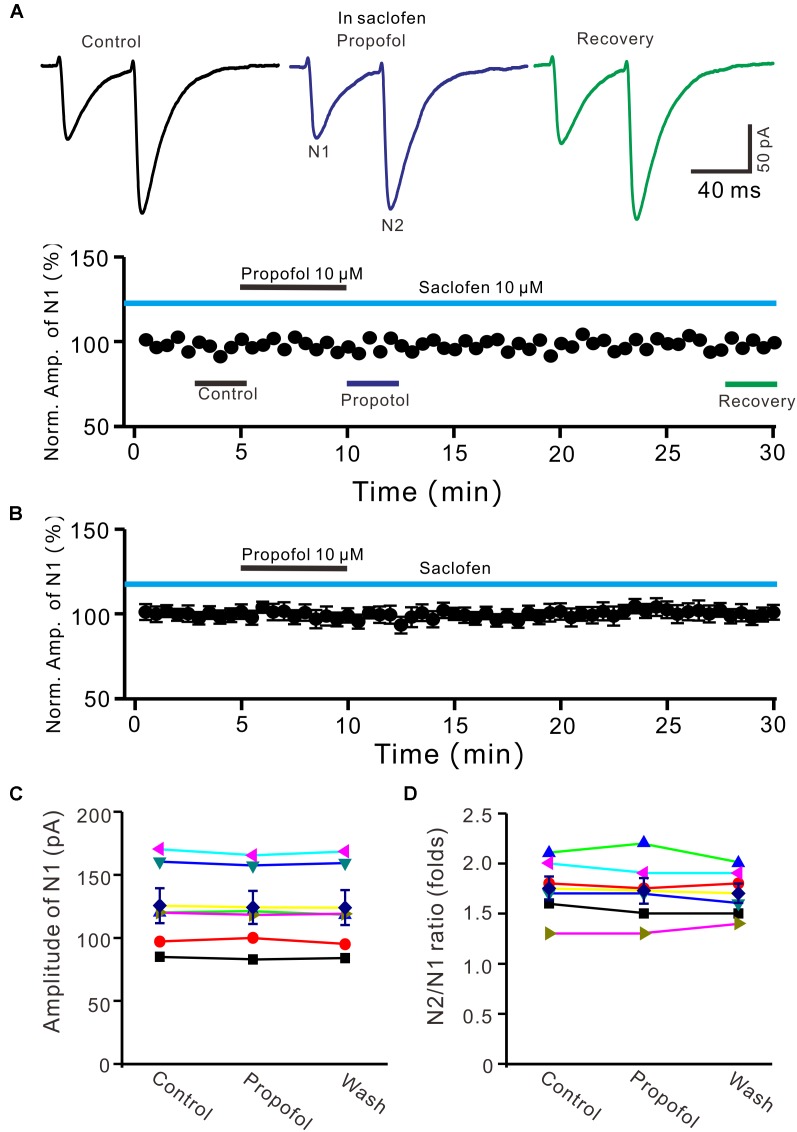
Blocking GABA_B_ receptor abolished the propofol-induced inhibition of PF-PC EPSCs. **(A)** Upper, representative traces showing PF-PC EPSCs elicited by paired-pulse stimulation under control (10 μM saclofen), propofol (10 μM propofol + 10 μM saclofen) and recovery (washout of propofol). Lower panel shows the time course of the propofol-induced changes in amplitude of N1 shown in upper panel. **(B)** Summary of data (*n* = 6) showing the time course of propofol-induced changes in amplitude of N1 in the presence of saclofen. **(C)** Individual and mean (±S.E.M) values showing the amplitude of N1 in the each treatment. **(D)** Individual and mean (±S.E.M) values showing the PPR in the each treatment.

It has been demonstrated that intravenous administration of propofol depressed the activity of nigral DA neurons, which was prevented by the selective GABA_B_-receptor antagonist (Schwieler et al., 2003). Therefore, we used a GABA_B_ receptor selective antagonist, saclofen (10 μM) to determine whether propofol-induced inhibition of PF-PC EPSCs occurred via activation of GABA_B_ receptor in the absence of GABA_A_ and glycine receptors activity. In the presence of saclofen, propofol failed to depress PF-PC EPSCs (Figures 4A,B). The mean amplitude of N1 was 125.3 ± 13.7 pA, which was similar to the control (124.1 ± 13.0 pA; *P* = 0.68; *n* = 6) (Figure 4C). The mean value of PPR was 1.73 ± 0.13 in the presence of propofol, which was similar to the control (1.75 ± 0.11; *P* = 0.73; *n* = 6) (Figure 4D). These results indicate that blockade of GABA_B_ receptors activity completely preventes the propofol-induced decrease in amplitude and increase in PPR of PF-PC EPSCs, suggesting that the propofol-induced depression of PF-PC EPSCs through activation of presynaptic GABA_B_ receptors in the absence of GABA_A_ and glycine receptors activity.

**FIGURE 5:**
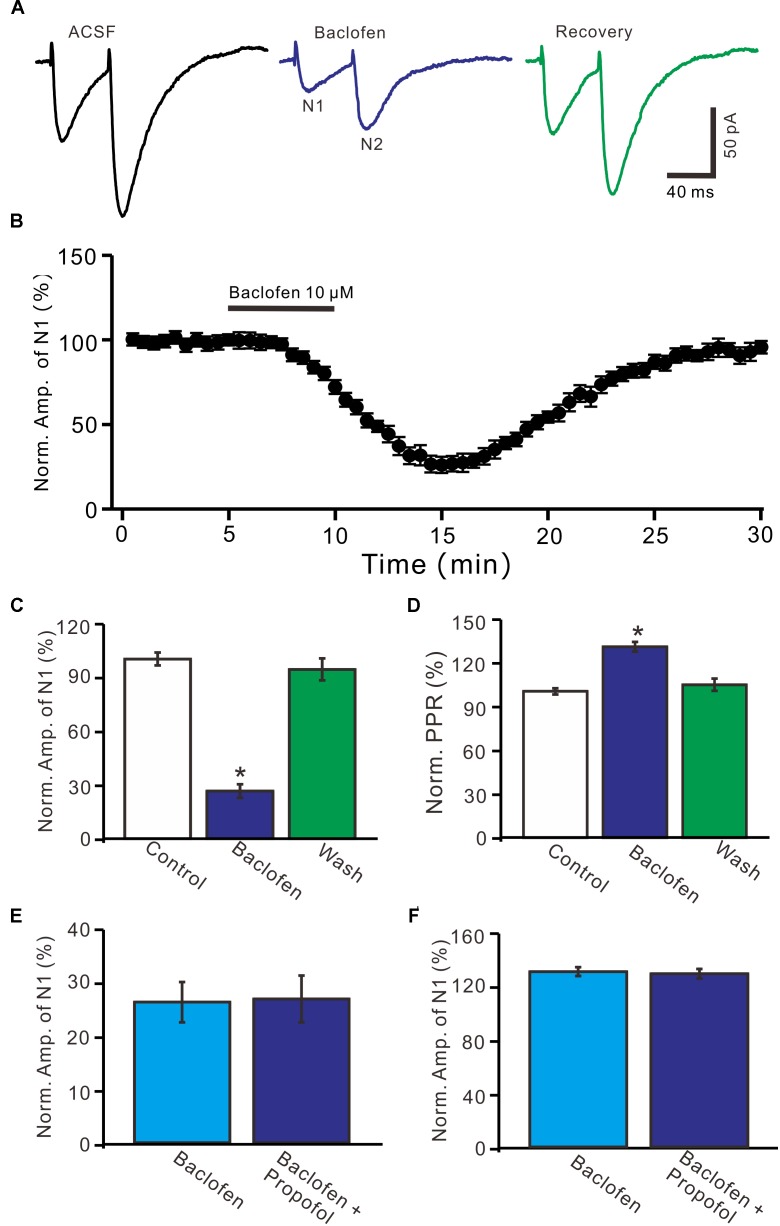
Effects of GABA_B_ receptor agonist, baclofen on amplitude and PPR of PF-PC EPSCs. **(A)** Representative traces showing PF-PC EPSCs elicited by paired-pulse stimulation under control, baclofen (10 μM baclofen) and recovery (washout of propofol). **(B)** Summary of data showing the time course of baclofen-induced changes in amplitude of N1. **(C)** Summary of data showing the normalized amplitude of N1 in the each treatment. **(D)** Pooled data (*n* = 6) showing the normalized PPR in the each treatment. **(E)** Summary of data showing the normalized amplitude of N1 in treatments of baclofen (10 μM) and a mixture of propofol (10 μM) and baclofen (1 μM). **(F)** Pooled data showing the normalized PPR in treatments of baclofen (10 μM) and a mixture of propofol (10 μM) and baclofen (10 μM). *n* = 6 in each group. ^∗^*P* < 0.05 vs control.

Further, we examined whether pharmacological activation GABA_B_ receptor induced a depression of PF-PC EPSCs. Application of baclofen (10 μM) induced a time-dependent depression of PF-PC EPSCs (Figures 5A,B). In the presence of baclofen, the normalized amplitude of N1 was 26.5 ± 3.8% of baseline (100 ± 3.6%; *P* < 0.0001; *n* = 6) (Figure 5C), and the normalized PPR value was 130.5 ± 3.3% of baseline (100 ± 2.1%; *P* = 0.002; *n* = 6) (Figure 5D). In addition, co-application of baclofen and propofol decreased amplitude of N1 to 27.1 ± 4.4% of baseline (100 ± 3.1%; *P* < 0.0001; *n* = 6), which was no significant difference with application of baclofen alone (26.5 ± 3.8% of baseline; *P* = 0.82; Figure 5E). The application of baclofen and propofol also increased PPR to 128.9 ± 3.6% of baseline (100 ± 2.7%; *P* = 0.003; *n* = 6), which was similar to application of baclofen alone (130.5 ± 3.3% of baseline; *P* = 0.76; Figure 5F). These results indicate that pharmacological activation of GABA_B_ receptors induces a decrease in amplitude and an increase in PPR of PF-PC EPSCs, as well overwhelms the effect of propofol on PF-PC EPSCs.

### The Propofol-Induced Depression of PF-PC EPSCs Was Required PKA

**FIGURE 6:**
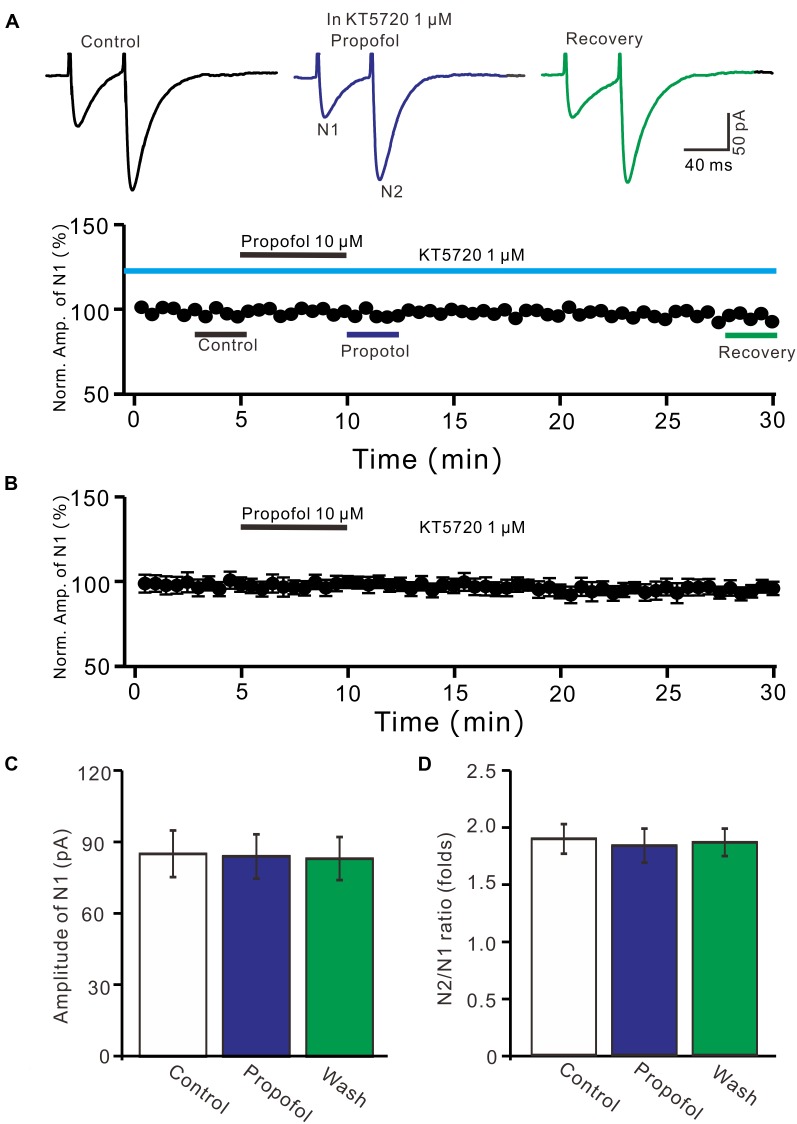
A specific PKA inhibitor, KT5720 completely prevented the propofol-induced inhibition of PF-PC EPSCs. **(A)** Upper, representative traces showing PF-PC EPSCs elicited by paired-pulse stimulation under control (1 μM KT5720), propofol (10 μM propofol + 1 μM KT5720) and recovery (washout of propofol). Lower panel shows the time course of the propofol-induced changes in amplitude of N1 shown in upper panel. **(B)** Summary of data (*n* = 5) showing the time course of propofol-induced changes in amplitude of N1 in the presence of a mixture of picrotoxin and KT5720. **(C)** Bar graph (*n* = 5) showing the amplitude of N1 in the each treatment. **(D)** Pooled data (*n* = 5) showing the PPR in the each treatment.

We further examined whether protein kinase A (PKA) was necessary for suppression of PF-PC EPSCs. In the presence of a specific PKA inhibitor, KT5720 there was no significant change in amplitude of PF-PC EPSCs following application of propofol (Figures 6A,B). In the presence of KT5720 and propofol, the mean amplitude of N1 was 84.9 ± 9.35 pA, which was no significant difference than that in control conditions (86.1 ± 9.8 pA; *P* = 0.71; *n* = 5) (Figure 6C), and the mean PPR value was 1.8 ± 0.15, which was similar to control conditions (1.9 ± 0.13-fold; *P* = 0.63; *n* = 5) (Figure 6D). These results indicate that inhibition of PKA abolishes the effects of propofol on amplitude and PPR of PF-PC EPSCs, suggesting that propofol-induced depression of PF-PC EPSCs through activation of presynaptic GABA_B_ receptors and PKA signaling in the absence of GABA_A_ and glycine receptors activity.

## Discussion

Our present results showed that propofol reversely decreased the amplitude of PF-PC EPSCs, accompanied with a significant increase in PPR in absence of GABA_A_ and glycine receptors activity. The propofol-induced changes in amplitude and PPR of PF-PC EPSCs were abolished by blockade of GABA_B_ receptors activity, as well by inhibition of PKA signaling pathway. These results indicate that application of propofol facilitate presynaptic GABA_B_ receptors, resulting in an inhibition of PF-PC synaptic transmission via PKA signaling pathway in mouse cerebellar cortex. Our results suggeste that propofol acts on presynaptic GABA_B_ receptors to modulate the PF-PC excitatory synaptic transmission *in vitro* in mice.

### Propofol Facilitates GABA_A_ and Glycine Receptors Mediated Inhibition in Cerebellar Cortical PCs

Clinically relevant concentrations of propofol significantly enhanced GABA-mediated inhibitory transmission in rat olfactory cortical neurons (Collins, 1988), and depressed neuronal spike firing via activation of GABA_A_ receptors (Jewett et al., 1992; Grasshoff and Antkowiak, 2004; Zhang et al., 2018). Propofol enhanced GABA_A_ receptors activity has been considered by extending open times and slowing desensitization of GABA_A_ channels (Bai et al., 1999, 2001; Kitamura et al., 2004), and decreased the facial stimulation-evoked spike firing activity of molecular layer interneurons through enhancement of GABA_A_ receptors activity (He et al., 2015). We recently found that simultaneously blocked GABA_A_ and glycine receptor and abolished the propofol-induced inhibition of the spontaneous SS activity of cerebellar PCs, indicating that propofol facilitated postsynaptic GABA_A_ and glycine receptors activity (Jin et al., 2015). In this study, we showed that propofol concentration dependently decreased the amplitude of PF-PC EPSCs in the absence of GABA_A_ and glycine receptors activity, suggesting that propofol depressed PF-PC synaptic transmission was independent on GABA_A_ and glycine receptors activity.

### Propofol Depressed PF-PC Synaptic Transmission Through Facilitation of GABA_B_ Receptor in Cerebellar Cortex

GABA_B_ receptors are G protein-coupled receptor, which exist on many glutamatergic terminals where they decrease the release of glutamate by inhibiting voltage-dependent Ca^2+^ channels and thus presynaptic calcium influx (Wu and Saggau, 1995, 1997). In cerebellar cortex, PF-PC synapses express GABA_B_ receptors which modulate neurotransmitter release and synaptic transmission (Dittman and Regehr, 1996, 1997; Orts-Del’Immagine and Pugh, 2018). Our present results showed that the propofol-induced a decrease in amplitude and an increase in PPR of PF-PC EPSCs were abolished by GABA_B_ receptor antagonist, indicating that the propofol-induced depression of PF-PC EPSCs through activation of presynaptic GABA_B_ receptors. Our present results are consistent with previous studies (Dittman and Regehr, 1996, 1997; Schwieler et al., 2003; Orts-Del’Immagine and Pugh, 2018), suggesting that activation of PF-PC GABA_B_ receptors depresses presynaptic release of glutamate. Dittman and Regehr (1996) demonstrated that activation of GABA_B_ receptors inhibited PF-PC synaptic transmission in rat cerebellar slices via modulation of presynaptic calcium channels (Dittman and Regehr, 1996) and voltage-gated calcium channels (Dittman and Regehr, 1997). Furthermore, activation of presynaptic GABA_B_ receptors induced inhibition of synaptic transmission at PF-molecular layer interneuron synapses in acute cerebellar slices from juvenile mice (Orts-Del’Immagine and Pugh, 2018). Moreover, intravenous administration of propofol depressed the activity of nigral DA neurons, which were prevented by the selective GABA_B_-receptor antagonist, suggesting that activation of GABA_B_ receptors might involve in the anesthetic action of propofol (Schwieler et al., 2003). Notably, our present results showed that GABA_B_ receptors agonist, baclofen, induced significant decrease in amplitude and an increase in PPR of PF-PC EPSCs, as well masked the effects of propofol on PF-PC EPSC, confirming that the propofol depressed of PF-PC EPSCs via activation of presynaptic GABA_B_ receptors.

On the other hand, we previously demonstrated that blockade GABA_B_ receptors activity failed to prevent the propofol-induced depression of SS firing rate of cerebellar PCs, indicated that GABA_B_ receptor did not involve in the propofol-induced inhibition of the PCs spike firing activity (Jin et al., 2015). Moreover, it has been demonstrated that 25–100 μM propofol failed to induce significant change in amplitude of PF-PC EPSCs, but high concentration propofol significant enhanced the amplitude of PF-PC EPSCs in rat acute cerebellar slices (Lee et al., 2018). The contradictory results between the previous study and our present results are considered by following reason. In cerebellar cortex, GABA_A_ receptors are expressed on the PF-PC presynaptic sites, and their activation induces increases in release probability at PF presynaptic sites and the amplitude of PF-PC EPSCs (Stell et al., 2007; Pugh and Jahr, 2011). Therefore, propofol increased amplitude of PF-PC EPSCs (Lee et al., 2018) might be related to activation of PF-PC presynaptic GABA_A_ receptor (Pugh and Jahr, 2011). However, our present results showed that propofol decreased amplitude and increased PPF of PF-PC EPSC in the absence of GABA_A_ receptors activity. Importantly, the propofol induced changes in PF-PC EPSCs were abolished by GABA_B_ receptor antagonist and mimicked by its antagonist, indicating that propofol activated presynaptic GABA_B_ receptors, resulting in a depression of PF-PC EPSCs, accompanied with an increase in PPR.

In addition, our results showed that inhibition of PKA abolished the effects of propofol on the PF-PC EPSCs, suggesting that propofol-induced depression of PF-PC EPSCs through PKA signaling pathway. It has been reported that GABA_B_ receptor agonist inhibited forskolin-induced enhancement of miniature excitatory postsynaptic currents, suggesting activation of GABA_B_ receptor depressed adenylyl cyclase level, while activation of GABA_B_ receptor induced inhibition of PF-stellate cell EPSCs and increase in PPR were prevented by application of adenylyl cyclase activator, forskolin, suggesting presynaptic GABA_B_ receptors are responsible for the inhibition of PF-stellate cell synaptic transmission through adenylyl cyclase-PKA signaling pathway (Orts-Del’Immagine and Pugh, 2018). Collectively, our results suggest that propofol couples to GABA_B_ receptor induces an inhibition of adenylyl cyclase-cyclic AMP signal-transduction, which might lead to down regulation of proteins phosphorylation on synaptic vesicles in presynaptic terminals of PF-PC synapses, resulting in a decrease in glulamate release from PF onto PCs.

## Conclusion

Taken together, our current investigation showed that propofol reversely decreased the amplitude and increase in PPR of PF-PC EPSCs. The propofol-induced changes in amplitude and PPR of PF-PC EPSCs were abolished by blockade of GABA_B_ receptors activity, as well by inhibition of PKA signaling pathway. Our results indicated that application of propofol facilitates presynaptic GABA_B_ receptors, resulting in an inhibition of PF-PC synaptic transmission via PKA signaling pathway in mouse cerebellar cortex.

## Author Contributions

RJ, C-PC, and D-LQ conceived and designed the experiments. F-LX, H-WW, and L-XC performed the experiments. C-PC, D-LQ, and H-WW analyzed the data. Y-HB contributed reagents, materials, and analysis tools. C-PC and D-LQ wrote the manuscript.

## Conflict of Interest Statement

The authors declare that the research was conducted in the absence of any commercial or financial relationships that could be construed as a potential conflict of interest.
